# PrismPlus: a mouse line expressing distinct fluorophores in four different brain cell types

**DOI:** 10.1038/s41598-018-25208-y

**Published:** 2018-05-08

**Authors:** Janak Gaire, Heui Chang Lee, Ray Ward, Seth Currlin, Andrew J. Woolley, Jason E. Coleman, Justin C. Williams, Kevin J. Otto

**Affiliations:** 1 Department of Neuroscience, University of Florida, Gainesville, FL USA; 2 J. Crayton Pruitt Family Department of Biomedical Engineering, University of Florida, Gainesville, FL USA; 3 Weldon School of Biomedical Engineering, Purdue University, West Lafayette, IN USA; 4 Department of Biomedical Engineering, University of Wisconsin-Madison, Madison, WI USA; 5 Department of Neurological Surgery, University of Wisconsin-Madison, Madison, WI USA; 6 Department of Pediatrics and Child Health Research Institute, University of Florida, Gainesville, FL USA; 7 Department of Materials Science and Engineering, University of Florida, Gainesville, FL USA; 8 Department of Neurology, University of Florida, Gainesville, FL USA; 9 Nanoscience Institute for Medical and Engineering Technology, University of Florida, Gainesville, FL USA; 10 Department of Electrical and Computer Engineering, University of Florida, Gainesville, FL USA

## Abstract

To screen the complex central nervous system (CNS) injury responses, we created a quadruple-labelled ‘PrismPlus’ mouse line with a genetically encoded distinct fluorescent tag in oligodendrocytes, microglia, neurons, and astrocytes. Cx3cr1-gfp and Prism mice originally developed by Jung *et al*., 2000 and Dougherty *et al*., 2012, respectively, were cross-bred. First, we confirmed the presence of fluorophores in appropriate cell types in PrismPlus mice. PrismPlus mice were then used to examine the cellular responses to brain implanted micro-devices. We observed an increase in microglial response at earlier time points as compared to 4 weeks, a progressive astrocytic response, and fewer neurons at the vicinity of an implanted device. These results are similar to what has been described in literature using other rodent strains, previously attainable only through time-consuming and variable immunohistochemistry methods. Finally, we demonstrate the compatibility of PrismPlus brain tissue with CLARITY, an advanced tissue clearing technique, opening the door to future thick tissue imaging studies. This report confirms PrismPlus transgenic fluorescence and highlights the utility of these mice to study CNS injuries. The work herein seeks to establish a novel transgenic mouse line to improve experimental scope, consistency, and efficiency for CNS researchers.

## Introduction

The successful demonstration of expression of green fluorescent protein (GFP), a fluorescent molecule derived from Jelly fish (*Aequorea victoria*), in bacteria (*E. coli*) and nematode (*C. elegans*) by Chalfie *et al*. in 1994, laid the foundation for the development of mice expressing GFP in all cells (popularly known as ‘green mice’), and many other reporter mouse lines expressing GFP in cell-specific targets^1–4^. Modifications to GFP and the discovery of several other fluorophores extended the palette of fluorescent proteins thus allowing diverse combinations of molecular tags^5–7^. Transgenic mouse lines with genetically encoded fluorescent proteins (FPs) in conjunction with fluorescent-based microscopy techniques offer considerable advantages to both *ex vivo* and *in vivo* studies.

*Ex vivo* fluorescent immunohistochemistry (IHC) has been a gold standard technique to observe cellular and subcellular details since first demonstrated by Coons *et al*. in 1941^8^. Typical IHC involves mechanical sectioning of a tissue into thin sections and staining with antibodies of interest; this process is labor-intensive and may lead to loss or distortion of relevant data. With the advancements in imaging techniques^9,10^ and emerging tissue clearing techniques, such as Scale^11^, SeeDB^12^, CLARITY^13^, ClearT^14^, and ScaleS^15^, we can obtain greater imaging depth thereby negating the need to physically section into thinner sections. However, tissue clearing methods do not facilitate antibody label penetration. Thicker samples require incubation in higher antibody concentration for several days and, usually, under non-physiological conditions such as strong detergents and high temperature. Together, this results in limited and/or inconsistent antibody labelling through the depth of the tissue, often leading to misinterpretation of the data. Aforementioned issues that are associated with both thin and thick tissue staining can be addressed by using a reporter system with genetically encoded FPs.

Reporter mouse lines with transgenic FPs have gained popularity in the field of neuroprosthetic research^16–18^. Brain implanted micro-devices exhibit performance decline post-implant; this loss of functionality is attributed to formation of a glial scar that is composed of reactive astrocytes and activated microglia/macrophages and a loss of local neurons^19–21^. However, studies aimed at characterizing the cellular responses do not always show consistent results. End-point histological assessment of the surrounding tissue, which involves physically sectioning and immunostaining steps, is a common practice, and given its shortcomings may contribute to this variability. More advanced techniques, such as Device-Capture Histology, have been employed to preserve the implant-surrounding tissue for intact analysis, but are limited by antibody penetration issues as the technique requires capturing thick tissue sections (~500 µm)^22–24^. Others have used transgenic mice with genetically encoded FPs to study the real-time biological response to implants, but were limited to a single FP^16^. Brain implanted devices elicit a complex cellular response that involves multiple cell types including microglia, astrocytes, oligodendrocytes, and neurons.

There are several mouse lines that express single fluorophores under a specific cell type, including a Cx3Cr1-gfp mouse line that expresses GFP in immune cells including microglia, macrophages, natural killer cells, and dendritic cells^3,4,7^. However, mouse lines expressing multiple fluorophores are constrained to mostly two cells due to allele problem and to address this, Dougherty *et al*., 2012 reported the development of a triple-labelled ‘Prism mouse’ line to demonstrate that multiple transgenes can be integrated into single locus^25^. The Prism mouse line expresses cyan fluorescent protein (CFP) under the control of myelin oligodendrocyte basic protein (Mobp) in oligodendrocytes, yellow fluorescent protein (YFP) under the control of snaptosomal associated protein KD25 (Snap25) localized to ribosomal proteins (L10a) in nucleolus and cytoplasm of neuronal soma, and *Discosoma sp*. red fluorescent protein (DsRed) under the control of aldehyde dehydrogenase 1 family, member L1 (Aldh1L1) expressed in astrocytes^25^. While serving as a significant resource to researchers, the Prism mouse line lacked the expression of a fluorophore in microglial cells, a cell type of primary interest in CNS injury and cortical implant research.

In this paper, we report the development and characterization of a ‘PrismPlus’ mouse line expressing distinct fluorophores in four different CNS cell types. This was achieved by carefully interbreeding Cx3cr1-gfp and Prism mice that were previously developed by Jung *et al*., and Dougherty *et al*., respectively^4,25^. We show that the offspring from the cross inherited transgenes from both mouse lines and express distinct fluorophores in four different CNS cells. Following validation, we also demonstrate the utility of this trangenic mouse line in studying the cellular response to a cortical implant injury model and its compatibility with an advanced tissue clearing method, CLARITY^13^.

## Results

### PrismPlus mouse line successfully inherited transgenes from parental lines

We obtained the Prism mouse lines (heterozygous) and the Cx3cr1-gfp (homozygous) from the Jackson Laboratory and first, established a colony of each lines. To generate a PrismPlus mouse line, homozygous Cx3cr1-gfp mice were paired with heterozygous Prism mice. This cross resulted in offsprings that were positive for both the transgenes from Cx3cr1-gfp and Prism mouse lines. Primers that were used to detect transgenes are listed in Table 1. Later, male and female offspring, positive for both transgenes were bred, to maintain and increase the colony size. Overall, the litter sizes were small (mostly 3–5) and subsequently the pups’ positive for both transgenes were low (below 20%). Although quantitative behavioral testing was not performed, we did not notice any abnormal behavior in PrismPlus mice.

**Table 1:** List of primers used for genotyping.

Strain/Transgenes	Mutant Primers	Internal Control Primers
Cx3cr1-GFP (Method I)	5′-CCCCTGAACCTGAAACATAAA-3′ 5′-CCCAGACACTCGTTGTCCTT-3′	5′-GTCTTCACGTTCGGTCTGGT-3′ 5′-CCCAGACACTCGTTGTCCTT-3′
Cx3cr1-GFP (Method II)	5′-GTTGTTAACTTGTTTATTGCAGC-3′ 5′-CCCAGACACTCGTTGTCCTT-3′	5′-CTTCTACGCCCTCGTCTTCA-3′ 5′-ACCCAGACACTCGTTGTCCT-3′
Prism (CFP transgene)	5′-TCGTGACCACCCTGACCTG-3′ 5′-GTGATATAGACGTTGTCGCTGATG-3′	5′-GGCAAAGGTGGAAATGAAGA-3′ 5′-CTCAGACCACACAGGGAATG-3′
Prism (DsRed transgene)	5′-CCCATGGTCTTCTTCTGCAT-3′ 5′-AAGGTGTACGTGAAGCACCC-3′	

**Figure 1:**
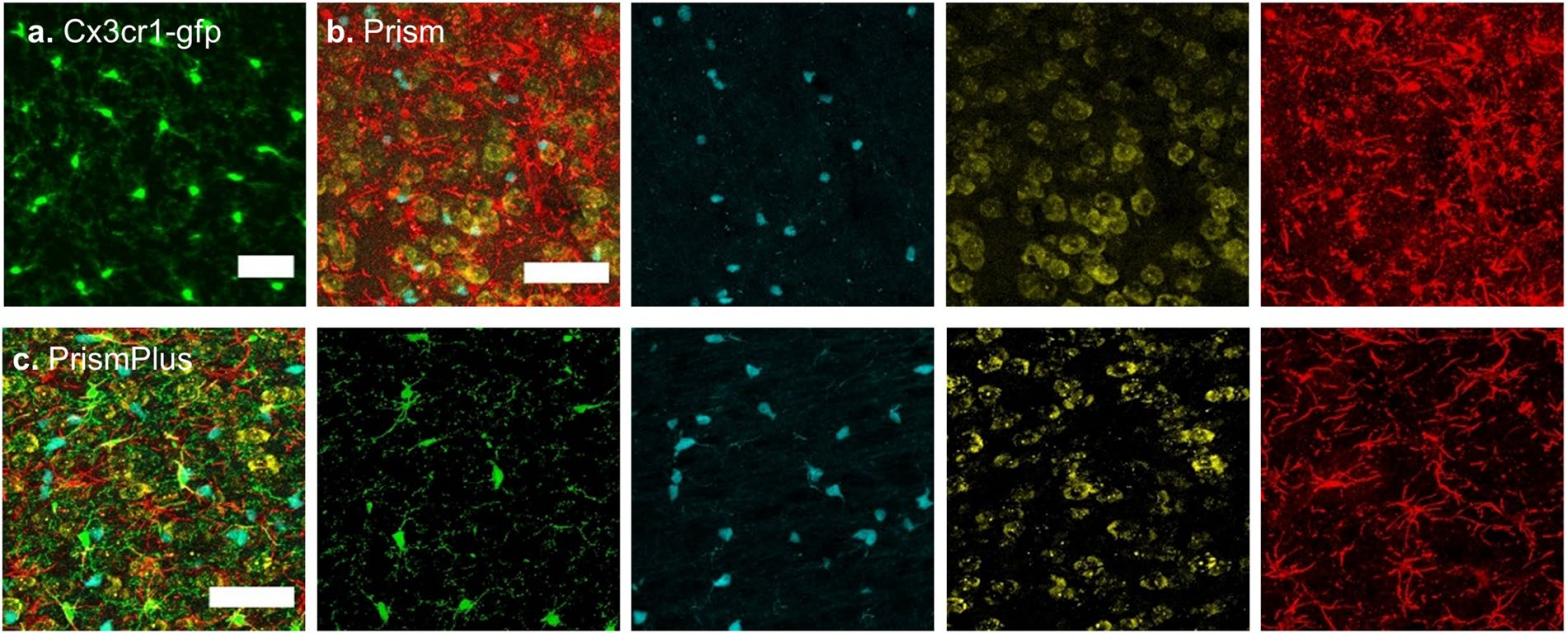
Confocal images of fluorophores expressed in four cell types in the cortex (approximately layer III-IV) of different mouse lines. (**a**) Green fluorescent protein (GFP) is expressed in microglia of Cx3cr1-gfp mouse. (**b**) Merged image of Cyan fluorescent protein (CFP) expressed in oligodendrocytes, yellow fluorescent protein (YFP) expressed in neurons and *Discosoma sp*. red fluorescent protein (DsRed) expressed in astrocytes of Prism mouse. (**c**) Merged image of CFP expressed in oligodendrocytes, GFP expressed in microglia, YFP expressed in neurons and DsRed expressed in astrocytes of quadruple-colored ‘PrismPlus’ mouse line. Single channel images from Prism and PrismPlus mouse lines are shown right to the merged images, b & c, respectively. Z-stack images of 20 microns thick sections were acquired either sequentially (**a**) or in lambda mode (**b**,**c**) using Zeiss LSM 710, 20x/0.8 NA objective and are presented as maximum projected intensity. Scale bar = 50 µm.

Following genotyping, phenotype validation was achieved by imaging the cortex of adult mice. Microglia (GFP) from Cx3cr1-gfp mice (Fig. 1a) and oligodendrocytes (CFP), neurons (YFP), and astrocytes (DsRed) from Prism mice (Fig. 1b) were present in the cortex of a PrismPlus mouse line (Fig. 1c) further confirming that the offspring positive for transgenes faithfully inherited cell-specific fluorophores from their parental lines. Images were collected spectrally, 9 nm interval, using lambda mode and separated via built in linear unmixing algorithm on a Zeiss LSM 710 microscope. Some GFP signal was noticed in YFP channels and were further processed; in particular, GFP signal from YFP channels were subtracted using built in tools in Zen software. Furthermore, microglial (GFP) and neuronal (YFP) morphology are visually distinguishable. The YFP neuronal signal was relatively dim in the cortex, however this was previously reported in the Prism mice as well^25^.

### CNS cells expressing distinct fluorophores in different brain regions co-localize with commercially available antibodies

**Figure 2:**
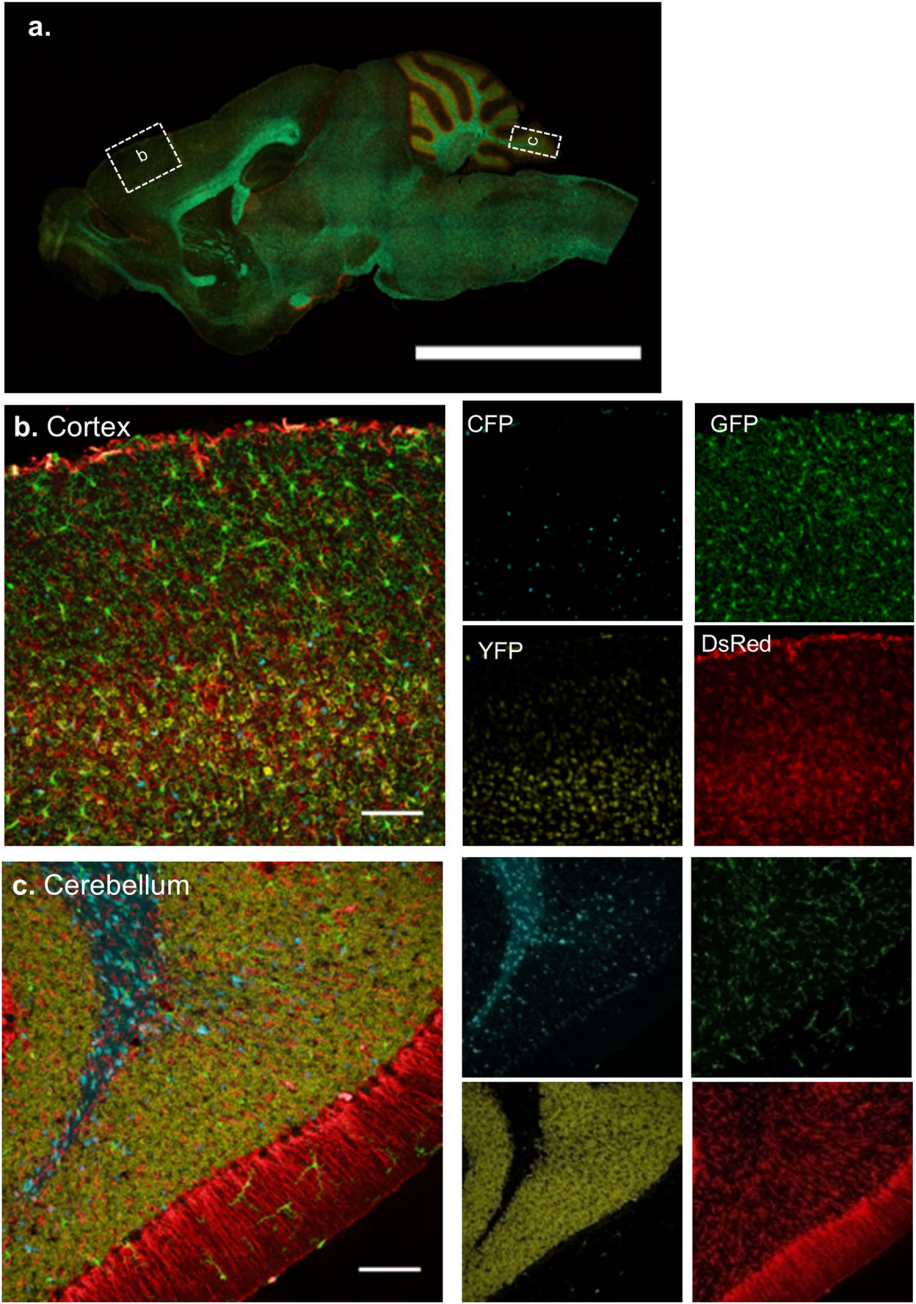
Expression of distinct fluorophores in CNS cells in different brain regions in a PrismPlus mouse line. (**a**) Epifluorescence image of a sagittal mouse brain section depicting approximate brain regions (dashed boxes) for images in b and c. CFP-expressing oligodendrocytes, GFP-expressing microglia, YFP-expressing neurons and DsRed-expressing astrocytes, and merged confocal images in cortex (**b**) and cerebellum (**c**) of a PrismPlus mouse. Single channel images of (**b**) and (**c**) are shown right to the merged images. Images are maximum projected intensity of 20 um thick section. Scale bar in a = 5 mm; b & c = 100 µm.

We observed the fluorescent cell distribution in different brain regions of the PrismPlus mice. An entire sagittal section of the brain (Fig. 2a.) shows the independent expression of cell-specific fluorophores across the entire brain of the PrismPlus mouse. To look at the cellular details, we focus on the cortex (Fig. 2b) and the cerebellum (Fig. 2c). CFP expressing oligodendrocytes are ubiquitous in deeper cortical layers and the white matter region of the cortex and cerebellum, especially at the densely myelinated region. GFP expressing microglia are evenly distributed and are presumably in the resting state in both regions. As expected, YFP expressing neurons are rare or absent in the molecular layer (layer I) as compared to deeper cortical layers, and densely packed in the cerebellar granule cell layer. DsRed expressing astrocytes are present in the outermost layer of the cortex (*glia limitans*), homogeneously distributed throughout the cortex, and astrocytes including bregmann glia in the cerebellum. We noticed bleed through of DsRed signals in other channels; however, the difference in morphology of the cell types helped guide signal separation by linear unmixing and look-up-table value selection.

**Figure 3:**
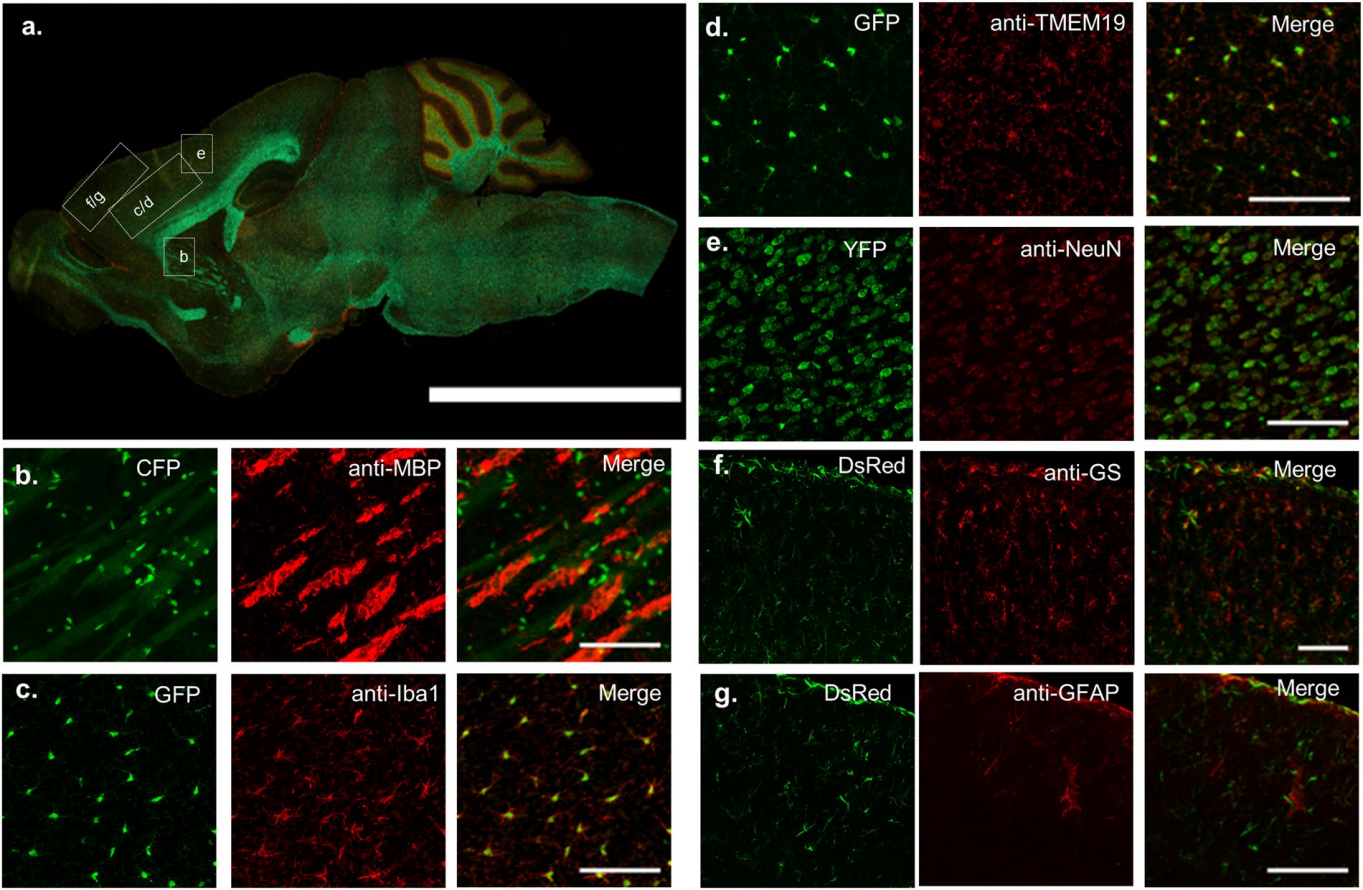
Co-localization of transgenic fluorophores with standard antibodies for different cell types. (**a**) Epifluorescence image of a sagittal mouse brain section illustrating approximate brain regions for images in b–g. (**b**) CFP-expressing oligodendrocytes co-occur with anti-MBP in white matter region of the brain. GFP-expressing microglia co-localized with anti- Iba1 (M1 = 0.824, M2 = 0.599) (**c**) and anti-TMEM19 (M1 = 0.806, M2 = 0.584) (**d**). (**e**) YFP-expressing neurons co-localized with NeuN (NeuN/YFP = 0.93, YFP/NeuN = 1.07). DsRed-expressing astrocytes co-localized with anti-glutamine synthetase (anti-GS) (M1 = 0.986, M2 = 0.692) (**f**) and anti-GFAP (M1 = 0.895, M2 = 0.573) (**g**). Different regions in the brain were selected to highlight specific cell’s distribution. Co-localization experiments was repeated at least on 3 different mice from different litters. M1 represents fraction of transgenic fluorophores overlapping immunostained cells and M2 represents fraction of immunostained cells overlapping transgenic fluorophores. Scale bar in a = 5 mm; b–g = 100 µm.

Next, we performed standard IHC using commercially available cell-specific antibodies to validate the utility of PrismPlus mouse line in studies where staining is carried out routinely to asses these specific CNS cell types. CFP fluorescing myelin oligodendrocyte basic protein co-occur with anti-MBP, a marker of oligodendrocytes, in white matter regions that are primarily composed of myelinated axons (Fig. 3b). Note that CFP is predominantly localized to the cell body nearby MBP which is cytoplasmic and membrane associated protein^26^. GFP fluorescing microglia co-localize with anti-Iba1 (M1 = 0.824, M2 = 0.599) and anti-TMEM19 (M1 = 0.806, M2 = 0.584) in tissue, markers that are used to identify microglial cells (Fig. 3c,d). Though the overlap coefficients were lower than 1 as indicated by M1 and M2 values, the ratio of GFP expressing microglia to antibodies Iba1 and TMEM19, different markers for microglia, is close to 1 at the cell body level as evident in Fig. 3c,d, respectively. Note, the GFP signal is more intense at the cell body (though processes are also identifiable) in contrast to anti-Iba1 which is expressed evenly across the entire microglia (cell body and processes). YFP fluorescing neurons co-localize with a neuronal nuclear stain, anti-NeuN, as evident in Fig. 3e. As with NeuN expression, YFP signal in PrismPlus is concentrated at the neuronal soma, especially at nucleolus and cytoplasm. The ratio of anti-NeuN to YFP positive neurons is 0.93 and YFP positive neurons to NeuN is 1.07 suggesting a strong overlap between NeuN immunostain and YFP expressing neurons. Finally, DsRed positive cells co-localize with astrocytic markers anti-glutamine synthetase (M1 = 0.986, M2 = 0.692) and anti-GFAP (M1 = 0.895, M2 = 0.573) as evident in Fig. 3f,g. The co-localization is most apparent at the outermost layer of cortex (glia limitans layer) and large vasculature. Additionally, as expected, we did not see the co-localization of commercially available antibodies with other fluorophores than its corresponding fluorophores i.e., anti-Iba1 and anti-TMEM19 with CFP, YFP and DsRed; anti- MBP with GFP, YFP and DsRed; anti-GFAP and anti-glutamine synthetase with CFP, GFP and YFP (data not shown).

### Cellular response to brain- implanted devices in PrismPlus mice

**Figure 4:**
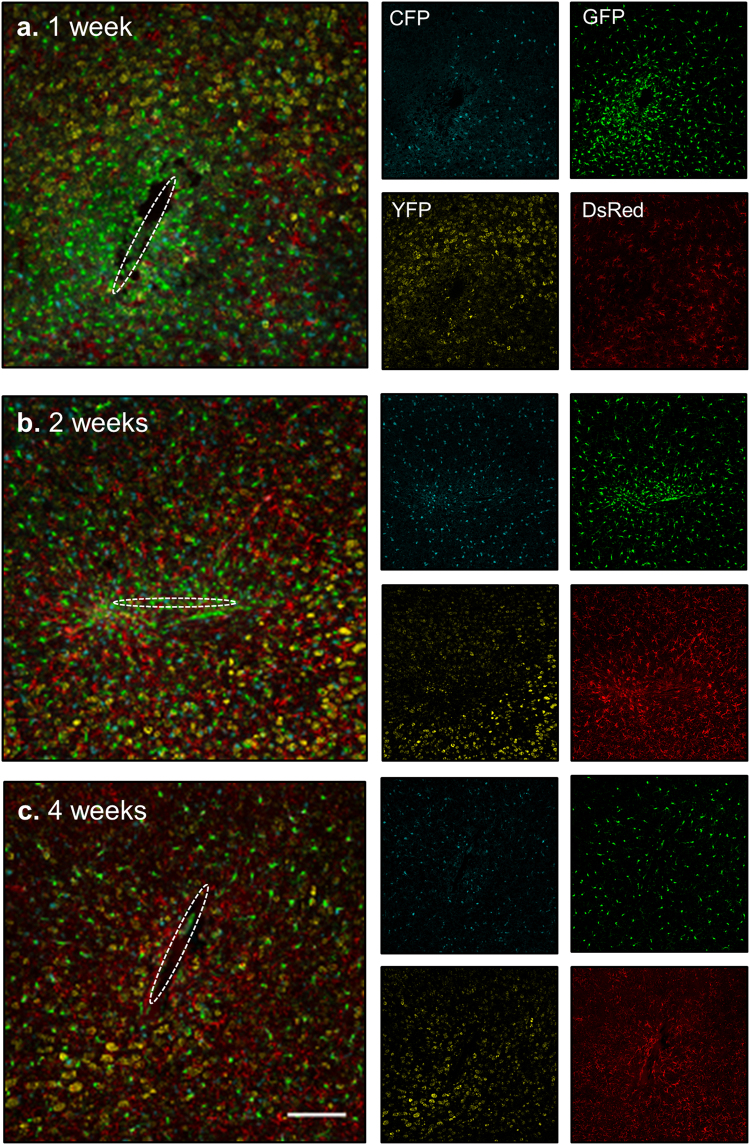
Cellular response to brain-implanted devices at multiple time points using PrismPlus mouse line. (**a–c**) Confocal images (merged) of cellular response to brain implanted devices at 1 week (**a**), 2 weeks (**b**) and 4 weeks (**c**) post implant. Dashed white ellipse represents the implant location. Merged images are separated into individual channels. Images are maximum projected intensity of 20 um thick sections and are approximately from the same depth (approximately 500 µm deep) in the cortex. Images in Fig. 4(a–c) were captured and processed using same settings. Objective: Plan-Apochromat 20x/0.8; Laser power: 405 nm: 5%, 488 nm: 6% and 543 nm: 0.2%. Scale bar = 100 µm.

**Figure 5:**
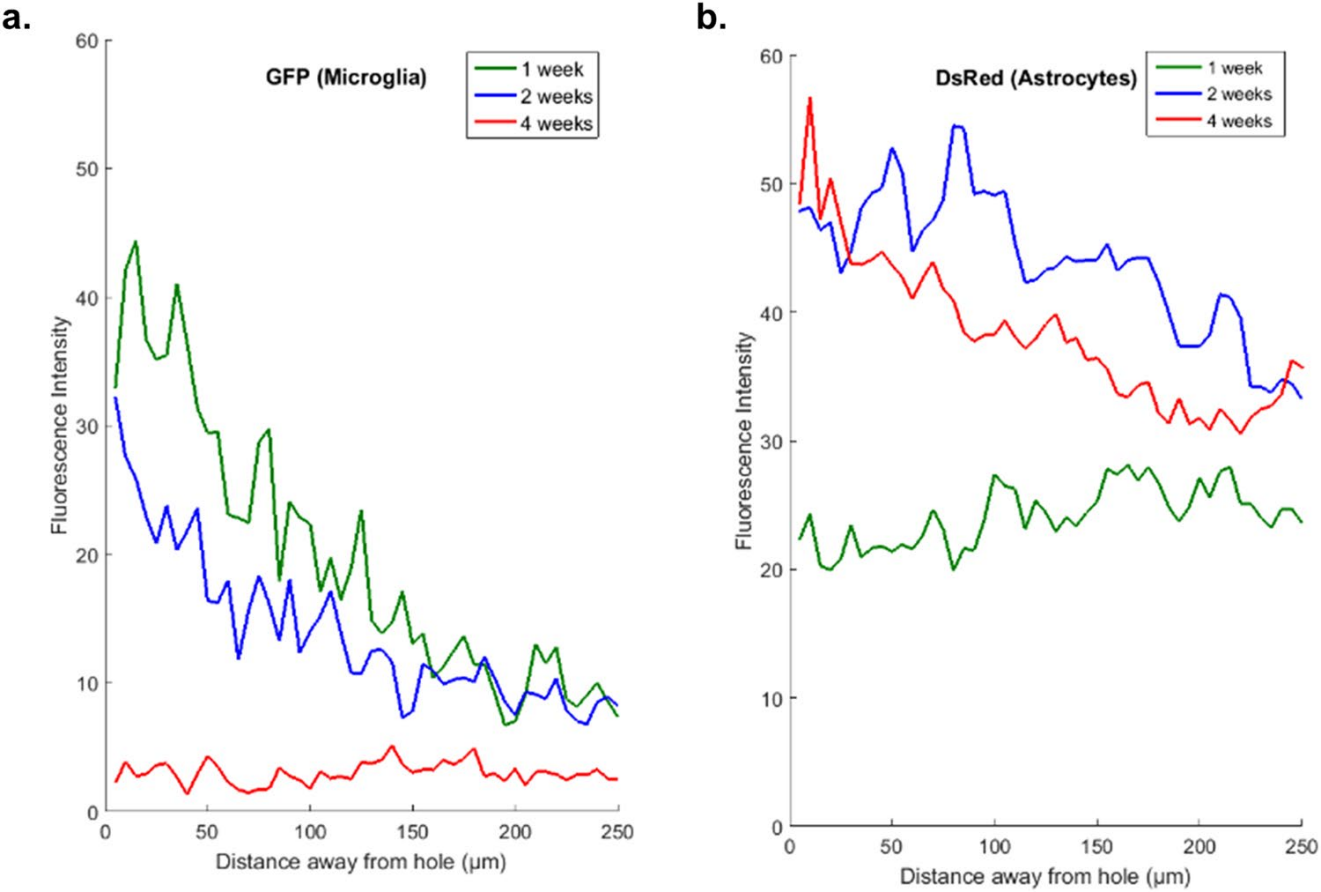
Fluorescence intensity profile of GFP expressing activated microglia/macrophages (**a**) and DsRed expressing astrocytes (**b**) as a function of distance (away from the ellipse drawn in Fig. 4) in PrismPlus mice implanted with “Michigan type” silicon device for 1 week (green), 2 weeks (blue) and 4 weeks (red). GFP and DsRed channels from images in Fig. 4 were used to generate the intensity profile.

One major goal of developing the PrismPlus mouse line was to allow researchers to quickly and consistently image essential cell types associated with CNS injury. For this study, we implanted silicon microelectrodes into the somatosensory cortex of PrismPlus mice and assessed the cellular response at three different time points (1 week, 2 weeks and 4 weeks; n = 1). We observed an increased microglial response, as indicated by an increase in number of GFP-positive cells around the implanted device at 1 week (Fig. 4a) and 2 weeks (Fig. 4b) compared to 4 weeks post-implant (Fig. 4c). Up to 150 µm away from the implant, fluorescent intensity of GFP was greater for 1 week implant as compared to 2 weeks and 4 weeks implant (Fig. 5a). The astrocyte response was a progressive, diffused response at earlier time points (1 week and 2 weeks) resulting in formation of an astroglial-sheath encapsulating the implanted device by four weeks. The DsRed fluorescent intensity was higher for 2 and 4 weeks as compared to 1 week (Fig. 5b) at the vicinity of implanted device. Though we noticed fewer YFP-positive cells up to 100 µm away from the implant site as compared to distant areas, we did not observe a complete ‘neuronal void’ surrounding an implant at any of the three different time points. There appeared to be a regular distribution of CFP positive oligodendrocytes surrounding the implanted devices out to 4-weeks.

### Fluorophores in PrismPlus mouse brain persist through the CLARITY clearing technique

**Figure 6:**
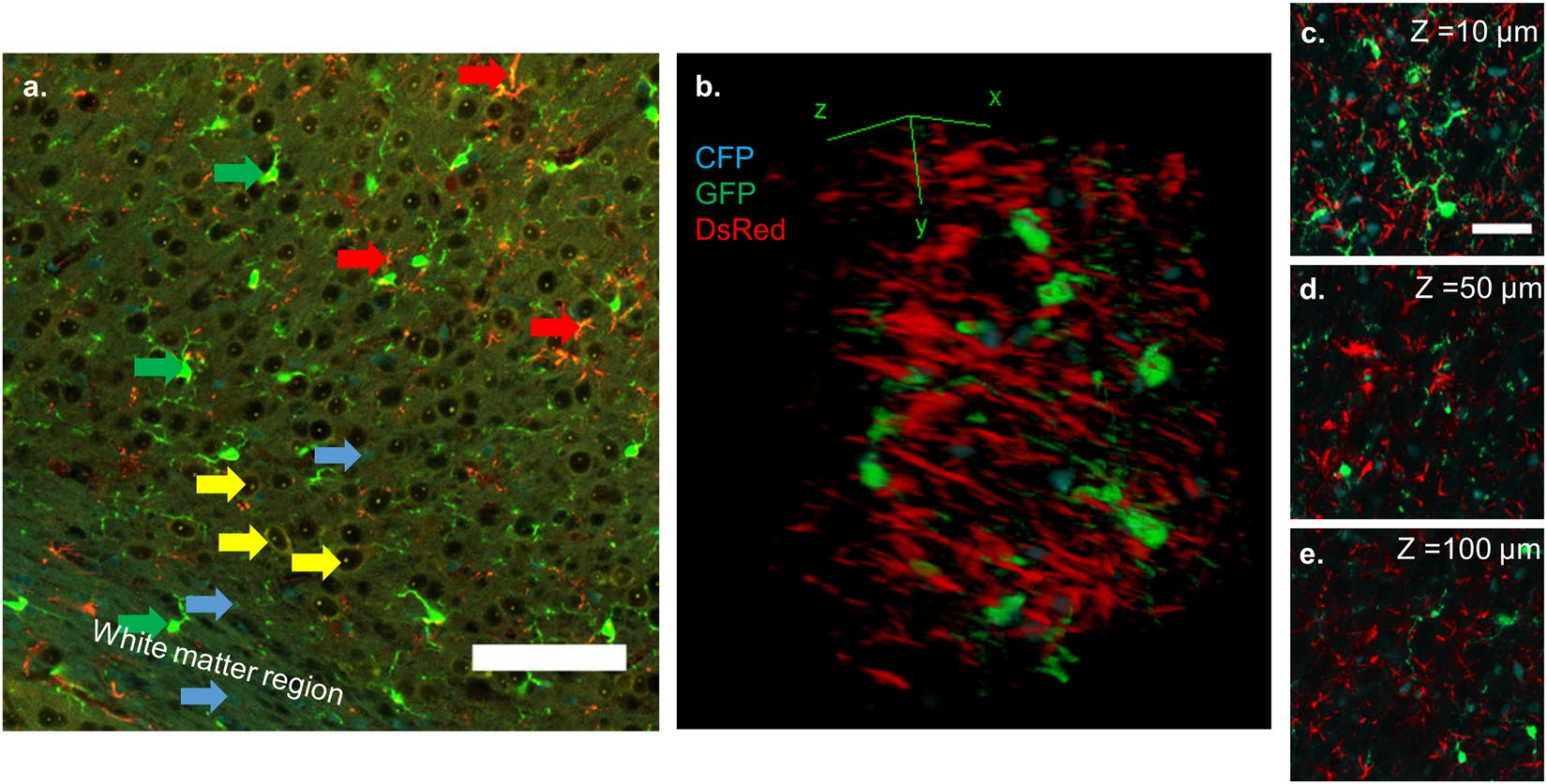
Retention of endogenous fluorophores in PrismPlus mouse brain tissue after CLARITY. (**a**) Confocal image (snapshot) of a cortex after active CLARITY showing that all fluorophores survived the clearing process. Note that YFP signal is prominent at the nucleolus and the nuclear envelope. Same colored arrow pointing at a particular cell type; Cyan arrow: CFP expressing oligodendrocytes, Green: GFP expressing microglia; Yellow:YFP expressing neurons, and Red: DsRed expressing astrocytes. (**b**) 3-D render view of 100 microns thick Z- stack of a PrismPlus brain (approximately layer IV-V in cortex) after CLARITY showing CFP expressing oligodendrocytes, GFP expressing microglia and DsRed expressing astrocytes. Figures C,D, and E represent 10 microns thick Z-stack projection at different Z-depths in Fig. B. Due to the punctate YFP, it was excluded in the Figures b,c,d, and e. Image in (**b**) was taken with a 20x objective with digital zoom of 2.0. Scale bar in a = 100 µm; c = 50 µm.

To assess the compatibility of fluorophores present in the brain tissue of PrismPlus mouse line with an advanced tissue clearing method, we cleared thick brain sections (>500 µm) with the CLARITY technique^13^. As evident in Fig. 6a, transgenic fluorophores were retained after the tissue clearing process and morphological hallmarks were preserved for each cell type. However, we noticed dimmer YFP signal in CLARITY cleared tissue than the tissue that did not go through the clearing procedure. Note the YFP signal (especially in cortex) is dimmer relatively to other fluorophores (Fig. 1c) even in brain tissue that did not go through clearing protocol. Yet, the nucleolus and the nuclear envelope of neurons are still identifiable in the cleared brain tissue (Fig. 6a). In addition, through Fig. 6(b–e), we demonstrate that a thick section can be imaged without any non-uniformity in fluorescent intensity, which is commonly seen in thick-tissue sections processed by IHC labelling^23^.

## Discussion

Transgenic animals with cell-type specific markers have become an invaluable tool in the field of biomedical sciences to study both persistent and highly dynamic events, such as injury response. However, we are restricted to mice expressing genetically encoded fluorophores in few cell types. In this report, we present the union of Cx3cr1-GFP mice with Prism mice to provide a more complete cellular overview of CNS cell types in a single animal. Following genotyping and verification of cell specific fluorophores, we explored the utility of PrismPlus line (i) in a cortical implant injury model, and (ii) in thick tissue imaging via an optical clearing technique.

Having multiple FPs with emission profiles close in the light spectrum in PrismPlus mouse tissue was advantageous for us as it allowed to stain with cell-specific antibodies utilizing Alexa dyes in far-red range. The overlap of transgenic FPs with comercially available antibodies ensured that the FPs were indeed expressed in correct cell types. Though the epitope target of antibodies are different than promoter under which the FPs are expressed, the degree of overlap between them illustrates that this mouse model can serve as an alternative to immunostaining where routine assessment of these cell-specific markers are performed. Furthermore, these results also demonstrates that five colour imaging can be easily achieved, if desired, with PrismPlus mouse tissue using a standard confocal microscope. However, the presence of CFP, GFP and YFP, in the same sample, whose emission peaks are in close proximity required special attetntion during image acquisition and post-processing of the imaging data. In our study, we accomplished this by either spectrally imaging (utilizing lambda mode of the confocal system) followed by linear unmixing or using narrow range filters, whenever sequential imaging was performed. While spectral imaging accomplished rapid collection of data, the cortical YFP signal, although morphologically discernable, was not as robust in our hands as compared to sequential scanning as evident in Figs 1c and 2b (YFP channel) *vs* Fig. 3e. A 543 nm beam splitter used during spectral scan prevented collection of all YFP signal. This issue might be less prominent with two-photon lasers providing tunable excitation with appropriate filter sets and spectral deconvolution algorithms for spectrally close fluorophores^27^.

One immediate application of PrismPlus mice is the study of neuroprosthetic device integration in CNS tissue, to assess the cellular response to brain implanted devices more consistently than immunostaining. CNS injury such as an injury model of cortical implant involves interplay between multiple cell types and it is difficult to interpret the complex relationship using monolabelling approach on neighboring sections. In Fig. 4, we demonstrate that the PrismPlus mouse recapitulates the cellular response to an injury model of cortical implants, which is typified by an acute injury response followed by an immunological foreign body response (FBR) to the chronically implanted microelectrodes within the CNS. The FBR involves multiple CNS cell types with each type following a stereotyped time course of migration and morphological changes^16,19,20,28,29^. A heightened microglial response, as suggested by both the increased number of GFP-positive cells and fluorescent intensity around the implant injury at earlier time points, are in line with previous findings in rodents^18,28,30,31^. The astrocytic response was progressive, transitioning from an initial diffuse response to the formation of an astroglial sheath by 4 weeks, similar to previous descriptions in rodent studies^19^. Though YFP signal was lower surrounding the implanted device, we did not observe loss of cells resulting in a large neuronal void near the implants, often referred as the ‘neuronal kill zone’^21^. Future experiments with longer time points may explain this phenomenon. Nonetheless, the presence of neuronal somas near the implants is not entirely suprising due to the variability often seen in histological study of micro-device implant integration^18,29^.

Among many factors including, but not limited to, heterogeneity of the cortex, suboptimal surgical procedure, and tissue processing steps that contribute to histological variations, IHC induced inconsistencies can be controlled through the use of PrismPlus mice. The consistency of the PrismPlus response relative to IHC processing of neuroprosthetic device integration suggest that it will be a valuable model for other injury models (e.g. stroke or traumatic brain injury) as well as neurodegenerative models (e.g. Parkinson’s Disease or Alzheimer’s Disease).

Reporter systems with genetically encoded FPs enable consistent labelling throughout thick tissue samples. Complimentary technology for the rapid imaging of optically cleared tissues, such as light sheet fluorescence microscopy, reduces volumetric imaging and reconstruction to hours, where two-photon microscopy can take days^32^. However, while the necessary clearing methods render the refractive index homogenous throughout the sample, they do not facilitate antibody penetration into thick tissue sections. Currently, the practice of large tissue clearing and imaging is limited by slow and limited diffusion of primary and secondary antibodies. We sought to confirm that the transgenic fluorescence signal did not fade as a result of the clearing protocol. In Fig. 6a, we demonstrate the retention of transgenic fluorophores following the advanced tissue clearing protocol. Though the nucleolar YFP signal was retained and the neuronal soma morphology is still identifiable after CLARITY protocol, we noticed dimmer YFP signal at the cytoplasm. The punctate, nucleolar YFP signal does not mark neuronal processes, however, like anti-NeuN, it allows reliable neuron population quantification in the imaging area. Though we were limited to 110 microns due to the limited working distance of the objective lens, greater imaging depth can be achieved by using objective lens with longer working distance utilizing either the confocal (single photon or multi-photon) or light sheet microscopy techniques. Furthermore, CLARITY compatible dyes that fluoresce at far-red region can be combined with improved histological methods utilizing PrismPlus mice to preserve the relevant histological information (surrounding the implanted device) that is otherwise lost with traditional device explant methodology^22–24,33^.

In conclusion, our data suggest that PrismPlus mouse line can provide a consistent and efficient platform for groups studying the FBR of brain-implanted devices or other CNS traumas, thereby addressing a significant sources of variability and inconsistency in this research area. We believe that this novel mouse line, in parallel with advanced microscopy and emerging tissue clearing techniques, will be instrumental in increasing efficiency of experiments in healthy CNS and injury models (cortical impact, stroke, etc.) where neuroinflammation plays a large role. We look forward to pairing PrismPlus mice with wide-view imaging modalities such as light sheet microscope, and with *in vivo* 2-photon window microscopy in longitudinal studies.

## Methods

All animal work involved in this study were carried out in accordance with National Institute of Health guidelines and were approved by Institutional Animal Care and Use Committee guidelines at the University of Florida.

### Mouse strains, colony maintenance, and genotyping

B6.129P-*Cx3cr1*^*tm1Litt*^*/*J (Stock number: 005582) and FVB- Tg(Prism) 1989Htz/J mice (Stock number: 018068) referred to as Cx3cr1-GFP and Prism, respectively were purchased from Jackson Laboratory (Bar Harbor, ME). Prism line was recovered from the cryopreserved stocks at The Jackson Laboratory facility. To maintain the Prism line colony, heterozygous male and female mice were bred. Prism lines were relatively poor breeders but responded well to being placed on high fat breeder chow and being provided mice huts to facilitate breeding. To generate a quadruple-colored ‘PrismPlus’ mouse line, mice homozygous for Cx3cr1-GFP (−/−) were crossed with heterozygous Prism (+/−) mice. For genotyping, tail-clips from litters were outsourced to GenoTyping Center of America (Ellsworth, ME). In brief, DNA was extracted using a Proteinase K solution and incubated overnight at 37 °C. Samples were diluted 1:50 in sterile water prior to PCR setup. Prism target was detected using four separate assays; two for cerulean and two for DsRed portion of the transgene. Cx3cr1-GFP target was also detected using four separate assays. Internal control and mutant primers used are listed in Table 1. The reactions were run using Quanta SYBR green mix on a LightCycler 480 under standard conditions with an anneal temperature of 60 °C followed by melt curve analysis. The CFP target had a melting peak of 86 °C and the internal control had a melting peak of 80 °C. The DsRed target had a melting peak of 86 °C. For Cx3cr1-GFP target, the melting peak of wild type target was 85.3 °C and the melting peak of mutant target was 85 °C.

### Microelectrode implantation

Following aseptic procedures, 249 µm wide and 15 µm thick planar ‘Michigan Array’ silicon microelectrodes were implanted in heterozygous PrismPlus mice (NeuroNexus, Ann Arbor, MI). Mice were induced with 3% isoflurane (Zoetis, Parsippany, NJ) in Oxygen, and maintained at 1% during surgery. Heating pad was placed underneath the animal to maintain the body temperature at 37 °C and vital signs were monitored with a pulse oximeter (Kent Scientific, Torrington, CT). A bolus of 1 mg/kg Meloxicam (Norbrook, United Kingdom) was given before surgery and up to 48 hours after the surgery. A small incision was made along the midline using fine scissors and underlying periosteum and connective tissue was removed to expose the skull. A small craniotomy, approximately 2 mm in diameter, was created over the barrel cortex using a micro drill, pausing and irrigating with saline frequently to avoid transient overheating of cortex. After the skull was thinned, it was gently lifted away using fine tipped tweezers (World Precision Instruments, Sarasota, FL). A microelectrode array was implanted 1.2 mm deep at 20 mm/s using an automated insertion system (Physik Instruments, Karlsruhe, Germany). After implantation, the exposed area of the electrode was clipped and a thin layer of Kwik-Sil (World Precision Instruments, Sarasota, FL) was applied to cover the craniotomy. Dental acrylic (Fusio Liquid Dentin, Pentron, Orange, CA) was then applied to cover the remaining area of exposed skull. A triple antibiotic (Actavis, NC) was applied around the surgical margins. Following the procedure, 0.5 ml of saline was injected subcutaneously, and the subjects were monitored until ambulatory. Mice were housed separately until sacrifice at 1, 2, and 4 weeks post implantation.

### Brain extraction, sectioning, and immunostaining

Mice were deeply anaesthetized and then transcardially perfused with 1x Phosphate Buffered Saline (PBS) followed by 4% paraformaldehyde (PFA) solution. The head was fixed overnight in 4% PFA at 4 °C followed by three PBS washes at room temperature (RT). The brain was extracted, cryoprotected in 30% sucrose solution at 4 °C overnight, then snap frozen in −40 °C 2-methylbutane (Sigma Aldrich, M32631-500 ML) for 2 minutes. Frozen brains were then mounted in Optimal Cutting Temperature (OCT) medium, sectioned into 20–25 µm thickness and stored on a slide glass (Superfrost Plus, Thermo Fisher Scientific, Waltham, MA) at −20 °C until further processing. For neural implant studies, implanted electrodes were explanted carefully after fixation and tissue was sectioned horizontally 1.2 mm deep or the tip of electrode track was visible. Slices were equilibrated to RT and washed three times in PBS for five minutes each to remove excess OCT. For co-localization experiments, slices were blocked in wash solution (1% vol/vol normal goat serum, 0.3% Triton-X in HEPES buffered hanks solution (HBHS)) for 2 hours at RT then incubated in primary antibodies (rabbit anti-MBP (Abcam, ab50390), rabbit anti-NeuN (EMD Millipore, ABN78), rabbit anti-Iba1 (Wako Chemicals, 019-19741), chicken anti-GFAP (EMD Millipore, AB5541), rabbit anti-TMEM19 (Abcam, ab209064), rabbit anti-glutamine synthetase (Abcam, ab73593)) overnight at 4 °C followed by three 15 minute washes in PBS. Slices were then incubated with secondary antibodies Alexa-fluor-633 goat anti-chicken or Alexa-fluor-633 goat anti-rabbit (Life Technologies) for 1 & 1/2 hours at RT. Slices were washed 3 times with PBS and cover-slipped using Vectashield mounting medium (Vector Laboratories, Burlingame, CA) or Fluoro-gel (Electron Micorscopy Sciences, Hatfield, PA) onto standard microscope slides. Slides were stored in the dark at 4 °C until imaged.

### Tissue processing for CLARITY

PrismPlus mice were transcardially perfused with PBS followed by a cocktail of hydrogel monomer solution^13^. The brain was extracted and incubated in monomer solution for 3–4 days and polymerized at 37 °C for 3 hours. Samples were washed with electrophoretic solution (Logos Biosystems, South Korea) and cut into >500 µm sections using a vibratome (Leica Microsystems). Brain sections were cleared using previously described CLARITY^13^. For active clearing, a temperature-controlled X-CLARITY chamber (Logos Biosystems, South Korea) was used. Once the samples were cleared they are incubated in 88% w/v Histodenz (Sigma Aldrich, St. Louis, MO) refractive index matching solution, prepared in 0.02 M phosphate buffer^34^.

### Image acquisition, processing, and analysis

All the images except the sagittal section were acquired using a Zeiss LSM 710 confocal microscope. Images were acquired spectrally (422 nm–724 nm; 9 nm interval) in lambda mode (20x objective, 0.8 NA) then linearly unmixed using reference spectra. Reference spectra were collected using the same laser settings and beam splitters from wild type (background autofluorescence), Cx3cr1-GFP (GFP channel), and Prism mice (CFP, YFP and DsRed channels) brain sections. Due to the overlap of emission profiles of spectrally closed FPs (CFP, GFP and YFP), even with linear unmixing, some GFP signal was present in nearby channels which was subtracted using built in software in Zen (Zeiss, Germany). For co-localization experiments, images were acquired sequentially with appropriate filter settings. CFP was excited with 405 nm and detected with 428–484 band pass filter, GFP was excited with 488 nm and detected with 486–524 band pass filter, YFP was excited with 514 nm and detected with band pass 525–554 filter, DsRed was excited with 555 nm and detected with long pass 555 and Alexa fluor were excited with 633 nm and detected with long pass 639. For neural implant studies, MINUTE v1.5 software was used to calculate intensity profile of GFP and DsRed^18,31,35^. An ellipse was drawn around the implant (dotted dash line) and fluorescence intensity was plotted as a function of distance away from implant surface. In addition to Zen software, open source FIJI was also used for image processing^36^.

Tile scans of an entire sagittal section was captured using Leica DMi8 microscope using a 10 ×/0.40 dry objective (Leica Microsystems, Wetzlar, Germany). The emission (Em.) and excitation (Ex.) filter cubes used for different fluorophores are as follows: CFP (Ex. 426–446, Em. 435–485), YFP (Ex. 490–510, Em. 520–550); Y3/RFP (Ex. 532–558, Em. 570–640) GFP (Ex. 450–490, Em. 500–550). Mosaic merge of acquired tiles was performed with linear blending and auto-stitching using Leica LAX software processing tools.

### Co-localization analysis

To get an estimate of overlap transgenic fluorophores (GFP and DsRed with their respective immunostains), we calculated Mander’s coefficients using Coste’s automatic threshold, M1 (fraction of transgenic fluorophores overlapping immunostained cells) and M2 (fraction of immunostained cells overlapping transgenic fluorophores), using JACOP plugin in ImageJ software^37^. For YFP and NeuN pair, three blinded individuals performed manual cell count on the gray scale images to obtain the ratio of NeuN stained cells to YFP positive cells.

### Data availability

The PrismPlus mouse line is now available from The Jackson Laboratory as JAX#031478. Further datasets will be available from the corresponding author on reasonable request.
